# UHPLC‐ESI‐QTOF‐MS^2^ analysis of *Acacia pennata* extract and its effects on glycemic indices, lipid profile, pancreatic and hepatorenal alterations in nicotinamide/streptozotocin‐induced diabetic rats

**DOI:** 10.1002/fsn3.2732

**Published:** 2022-01-19

**Authors:** Hui Shao, Minmin Xiao, Zheng Zha, Opeyemi Joshua Olatunji

**Affiliations:** ^1^ Department of Clinical Laboratory East China Normal University Affiliated Wuhu Hospital Wuhu China; ^2^ Faculty of Traditional Thai Medicine Prince of Songkla University Hat Yai Thailand

**Keywords:** *Acacia pennata*, diabetes mellitus, glucose intolerance, oxidative stress, polyphenols, streptozotocin

## Abstract

Diabetes mellitus (DM) is a chronic disorder associated with severe metabolic derangement and comorbidities. The constant increase in the global population of diabetic patients coupled with some prevailing side effects associated with synthetic antidiabetic drugs has necessitated the urgent need for the search for alternative antidiabetic regimens. This study investigated the antidiabetic, antioxidant, and pancreatic protective effects of the *Acacia pennata* extract (APE) against nicotinamide/streptozotocin induced DM in rats. The antidiabetic activity of APE was evaluated and investigated at doses of 100 and 400 mg/kg body weight, while metformin (150 mg/kg bw) was used as a standard drug. APE markedly decreased blood glucose level, homeostatic model assessment for insulin resistance, serum total cholesterol, triglycerides, low‐density lipoprotein, blood urea nitrogen, creatinine, alanine transaminase, aspartate transaminase, and alanine phosphatase levels. Additionally, treatment with APE increased the body weight, serum insulin concentration, and high‐density lipoprotein. Moreover, activities of pancreatic superoxide dismutase, catalase, and glutathione peroxidase were increased, while the altered pancreatic architecture in the histopathological examination was notably restored in the treated rats. Ultra‐high performance liquid chromatography combined with electrospray ionization quadrupole time‐of‐flight mass spectrometry (UHPLC‐ESI‐QTOF‐MS) analysis of APE showcases the prevailing presence of polyphenolic compounds. Conclusively, this study showed the beneficial effects of the *Acacia pennata* in controlling metabolic derangement, pancreatic and hepatorenal dysfunction in diabetic rats.

## Graphical abstract

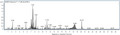

## 1 INTRODUCTION

Diabetes mellitus (DM) is one of the most common metabolic diseases that cut across all age groups and ethnicity. DM is clinically defined as hyperglycemia and it is accompanied by severe deteriorating comorbidities including diabetic nephropathy, neuropathy, reproductive dysfunction, foot ulcer, retinopathy, cardiovascular diseases, depression, and Alzheimer's disease (Makinde et al., 2020; Song et al., 2021). Statistically, 463 million adults were diabetic in 2019 and the global population of diabetes will increase to 700 million by 2045, and 90% of these reported cases is attributed to type 2 diabetes (IDF Report, 2019). In addition, several millions of people living with diabetes are undiagnosed or are not even aware of their diabetic status (International Diabetes Federation, 2019). Aside the public health burden that DM poses, the significant economic burden associated with DM is very huge. Over 760 billion dollars were expended on diabetes, and it is estimated that 845 billion dollars would be utilized globally for diabetes health‐related expenditure (Williams et al., 2020). A relatively sizeable population of people living with diabetes are low‐income earners or live in developing nations, where the huge financial burden associated with the treatment of DM is almost not affordable. Furthermore, the antidiabetic drugs currently in clinical use are either not affordable or accessible to most diabetic patients, in addition to the various side effects associated with these drugs (Olatunji et al., 2018; Song et al., 2021). These limitations further increase the morbidity and mortality rates associated with DM. As such, the search for affordable, easily accessible, safer, and indigenous antidiabetic regimens is imperative.

In many countries across the world especially in Asia and Africa, the use of medicinal plants or herbs constitutes a vital part in primary health care due to either religious or cultural beliefs. Specifically, numerous studies have highlighted the relevance of medicinal plants in the treatment of DM and DM‐associated comorbidities (Andrade et al., 2020; Farzaei et al., 2017). Additionally, the perceived safety and efficacy index of medical plants has increased their relevance across the globe. *Acacia pennata* (family: Mimosaceae) is a perennial herb vegetable domiciled in several countries including China, Thailand, Myanmar, and Bangladesh (Kim et al., 2015). In Thailand, the plant is widely used in Thai cuisine. *A. pennata* is rich in terpenoids and flavonoid glycosides. Traditionally, the plant is used alone or in combination with other plants for treating diabetes, cough, fever, headaches, snake bites, scorpion stings, and rheumatism (Andrade et al., 2020). Regarding pharmacological studies, *A. pennata* has been reported to possess significant antinociceptive, antioxidant, antimicrobial, anti‐inflammatory, α‐glucosidase and α‐amylase inhibition as well as anti‐Alzheimer's properties (Andrade et al., 2020; Dongmo et al., 2005, 2007; Lomarat et al., 2015). However, studies relating to the antidiabetic effects of the plant are still lacking and there are no scientific reports available to validate its traditional use as an antidiabetic regimen. Therefore, this study investigated the antidiabetic, antihyperlipidemic, and antioxidant effects of *A. pennata* extract in diabetic rats.

## 2 MATERIALS AND METHODS

### 2.1 Plant material

The aerial part of *A. pennata* was purchased from a local vegetable market in Hat Yai (Songkla, Thailand) in June 2020, and the identification was performed at the Faculty of Thai Traditional Medicine, Prince of Songkla University, Thailand. A reference sample (#FBRSVP0000270438) was kept at the herbarium of the faculty.

### 2.2 Drugs and chemicals

Metformin hydrochloride, nicotinamide, and streptozotocin were purchased from Alfa Aesar. All other chemicals and reagents used were of analytical grade.

### 2.3 Preparation of *Acacia pennata* extract (APE)

The plant was thoroughly cleaned under running tap water and oven dried for 4 days. The dried aerial part of the plant was pulverized with a mechanical grinder. Five hundred grams of the powdered sample was macerated in 70% ethanol at a solvent/solute ratio of 10:1 (v/w) for 24 h on a shaker. The extracted solution was filtered and concentrated to 30% of the original solution using a rotary evaporator. The resulting solution was dechlorophyllized using the sedimentation procedure (Olatunde, Benjakul et al., 2021; Olatunde, Tan et al., 2021; ). Briefly, the solution of the extract was kept overnight at 4°C. Thereafter, the sample was centrifuged at 10,000 *g* for 30 min at 4°C. The supernatant was collected and lyophilized. The light brown extract was termed “APE” and stored until further use.

### 2.4 UHPLC‐DAD‐ESI‐QTOF‐MS profiling of *Acacia pennata* extract

UPLC‐ESI‐Q‐TOF‐MS analysis of APE was performed by solubilizing 50 mg of APE powder in 1 ml of 50% aqueous methanol. The resulting solution was centrifuged and the clear supernatant obtained was subjected to UHPLC‐ESI‐QTOF‐MS analysis using the previously reported protocol (Olatunji et al., 2021).

### 2.5 Animals, establishment of diabetes, and treatment

Specific pathogen‐free male rats (7 weeks old; 185 ± 20 g) were used for the in vivo antidiabetic experiment. The animals were accommodated in stainless cages and fed with standard rat chow and water ad libitum. Additionally, the animals were maintained at a temperature of 22 ± 2°C, relative humidity of 55 ± 5%, and a 12 h light and dark cycle to simulate natural rhythm. The experimental protocol used in this study conformed to the guidelines of the National Institutes of Health (NIH, revised 1979) and was approved by the Ethics Committee of the East China Normal University Affiliated Wuhu Hospital (approval number, Wuhuey/2021/0926). The animals were acclimatized to the experimental environment for 1 week. Thereafter, type 2 diabetes mellitus was induced in overnight fasted rats by administering an intraperitoneal injection of 150 mg/kg of nicotinamide 30 min before administering streptozotocin (65 mg/kg, i.p.; Abdel Aziz et al., 2020; Ojuade et al., 2021). Seventy‐two hours poststreptozotocin administration, the fasting blood glucose (FBG) level of the overnight fasted rats was determined using Accu‐Chek guide glucometer to confirm the establishment of DM. Rats with FBG concentration above 250 mg/dl were adjudged diabetic and incorporated into the study. Diabetic rats were then randomly assigned into four groups of six rats each as follows:

Diabetic control rats (DMR): treated with normal saline.
Positive control rats (PCR): treated with 150 mg/kg of metformin.
APE‐1: diabetic rats administered 100 mg/kg of APE.
APE‐2: diabetic rats administered 400 mg/kg of APE.

Six healthy nondiabetic rats were designated as normal control (HNR) and were administered normal saline. The doses of the extract and metformin used were adopted from previous studies (Dongmo et al., 2005; Xiang et al., 2021). All the groups were administered their respective treatments once a day per os for 4 weeks. A weekly measurement of the body weight and FBG concentration was performed, while the water and food intake was determined on a daily basis.

### 2.6 Posttreatment intraperitoneal glucose tolerance test (IPGTT)

After the treatment, the rats were fasted overnight and a solution of 2 g/kg glucose was intraperitoneally injected into each rat from all the experimental groups. The blood glucose concentration was determined from the blood sample taken from the tail vein of each rat at an interval of 30 min for 120 min (Makinde et al., 2020).

### 2.7 Animal sacrifice and biochemical analysis

The rats were anesthetized under thiopental sodium and blood was collected via cardiac puncture, centrifuged to obtain the serum that was used for the determination of lipid profiles, hepatorenal biomarkers, and insulin levels. The pancreas was excised after animal sacrifice for histopathology using routine hematoxylin and eosin (H&E) procedures.

### 2.8 Determination of serum biochemical parameters

The serum obtained from the blood samples after centrifugation was used for the determination of serum lipids (triglycerides (TG), total cholesterol (TC), LDL‐C, and HDL) and hepatorenal biomarkers (ALT, AST, ALP, creatinine, BUN, and uric acid) using an automated chemistry analyzer (Zhang et al., 2020).

### 2.9 Determination of hemoglobin A1c and fasting plasma insulin

Hemoglobin A1c (HbA1c) and fasting plasma insulin (FINS) were determined with enzyme‐linked immunosorbent assay (ELISA) kits from the Nanjing Jiancheng Bioengineering Institute (China) according to the protocols of the manufacturer, while HOMA‐IR was determined using the formula stated below:

HOMA‐IR = (FBG × FINS)/22.5

### 2.10 Pancreas histological assessment

The pancreas was preserved in 10% neutral buffered formalin solution was used for the histological assessment using routine standard hematoxylin and eosin (H&E) procedures, as previously described (Olatunji et al., 2021).

### 2.11 Oxidative stress biomarkers

Oxidative stress parameters including superoxide dismutase (SOD), catalase (CAT), glutathione peroxidase (GPx), and malonaldehyde (MDA) levels were determined with biochemical kits from Nanjing Jiancheng Bioengineering Institute following the manufacturer's instructions (Gao et al., 2021; Olatunji et al., 2017).

### 2.12 Statistical analysis

GraphPad Prism 5.0. was used for statistical analysis, and data were presented as mean ± SD (*n* = 6). Statistical significance was assessed using one‐way analysis of variance (ANOVA) followed by a Dunnett post hoc test for comparison between experimental groups. The value of *p* < .05 was considered significant.

## 3 RESULTS

### 3.1 Identification of secondary metabolites in *Acacia pennata* extract

**FIGURE 1:**
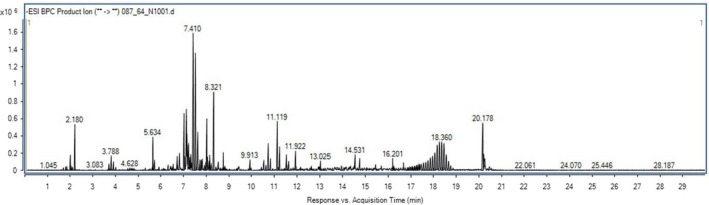
Total ion chromatograms of *Acacia pennata* extract (APE) using UHPLC‐DAD‐ESI‐QTOF‐MS

The identification of the bioactive metabolites in APE was carried out using UHPLC‐ESI‐QTOF‐MS/MS (Figure 1). As shown in Table 1, APE has a robust polyphenolic content ranging from simple phenolic compounds like trolox, to flavones such as baicalein and luteolin. Several flavonoid/phenolic glycosides were identified in the UHPLC‐ESI‐QTOF‐MS/MS analysis of APE, including cynaroside A, rutin, glucocaffeic acid, patuletin 3‐rhamnoside‐7‐(3''',4'''‐diacetylrhamnoside), Saponarin, luteolin 3'‐methyl ether 7,4'‐dixyloside, isovitexin, rhamnetin 3‐rhamnoside, quercetin 3‐rhamnoside‐3'‐sulfate, and apigenin 7‐methyl ether 4'‐glucoside. Aside the aforementioned phenolic/polyphenols mentioned above, other secondary metabolites identified in APE include nucleosides; namely 8‐hydroxyadenine, adenine, and isoguanosine. Pyrrolizidine alkaloids including usaramine and rosmarinine were also identified. 8‐Deoxy‐11,13‐dihydroxygrosheimin (sesquiterpene lactone) and oleandolide (macrolide)were also tentatively identified in APE.

**TABLE 1:** Compounds identified in *Acacia pennata* extract (APE) by the UHPLC‐ESI‐QTOF‐MS/MS analysis

No	Rt (min)	Accurate mass (*m/z*)	Calculated mass	Base peak (*m/z*)	Score (DB)	Predicted formula	Compound identity
1	2.251	191.0562	192.0635	191.0563	99.64	C_7_H_12_O_6_	Quinic acid
2	2.351	643.1718	644.1797	643.1714	63.9	C_34_H_32_FeN_5_O_5_	Nitrosyl‐heme
3	3.807	134.0473	135.0546	134.047	87.28	C_5_H_5_N_5_	Adenine
4	4.109	150.0418	151.0493	133.0155	78.88	C_5_H_5_N_5_O	8‐Hydroxyadenine
5	5.138	282.0841	283.0913	283.2639	92.9	C_10_H_13_N_5_O_5_	Isoguanosine
6	5.188	142.0507	143.0578	142.0504	82	C_6_H_9_NO_3_	Trimethadione
7	5.422	243.0508	244.0581	111.0448	99.2	C_10_H_12_O_7_	1‐O‐Galloylglycerol
8	6.117	387.0722	388.0785	387.0719	65.39	C_19_H_16_O_9_	Urolithin B 3‐O‐glucuronide
9	6.142	301.0564	302.0638	301.056	91.75	C_13_H_10_N_4_O_5_	Nicarbazin
10	6.318	323.1348	324.1421	323.1347		C_19_H_20_N_2_O_3_	p‐Hydroxyphenylbutazone
11	6.493	353.0876	354.0949	191.0557	98.26	C_16_H_18_O_9_	Chlorogenic acid
12	6.519	443.192	444.1993	443.1918	98.27	C_21_H_32_O_10_	Cynaroside A
13	6.744	285.0616	286.0688	285.0612	99.89	C_12_H_14_O_8_	Uralenneoside
14	6.82	609.1468	610.154	609.146		C_27_H_30_O_16_	Rutin
15	6.945	263.0772	264.0846	245.0666	97.16	C_10_H_16_O_8_	3‐Hydroxy−4‐butanolide
16	6.958	581.1516	582.1586	581.1508	98.53	C_26_H_30_O_15_	Norrubrofusarin 6‐beta‐gentiobioside
17	6.97	341.0877	342.0953	341.09	90.99	C_15_H_18_O_9_	Glucocaffeic acid
18	7.07	707.1829	708.1902	353.0873	99.39	C_32_H_36_O_18_	Patuletin 3‐rhamnoside−7‐(3''',4'''‐diacetylrhamnoside)
19	7.184	593.1515	594.1588	593.1511	98.38	C_27_H_30_O_15_	Saponarin
20	7.259	387.1665	388.1737	387.166	97.86	C_18_H_28_O_9_	2‐[4‐(3‐Hydroxypropyl)−2‐methoxyphenoxy]−1,3‐propanediol 1‐xyloside
21	7.422	563.1412	564.1484	563.1407	98.4	C_26_H_28_O_14_	Luteolin 3'‐methyl ether 7,4'‐dixyloside
22	7.748	447.0933	448.1006	447.0928	98.95	C_21_H_20_O_11_	1,2,6,8‐Tetrahydroxy−3‐methylanthraquinone 2‐O‐b‐D‐glucoside
23	7.874	327.051	328.0582	327.0506	98.83	C_17_H_12_O_7_	Aflatoxin M4
24	7.949	367.1034	368.1107	191.0553	97.56	C_17_H_20_O_9_	3‐O‐Caffeoyl−4‐O‐methylquinic acid
25	8.05	337.0931	338.1003	191.0557	98.44	C_16_H_18_O_8_	Hydrojuglone glucoside
26	8.20	431.0982	432.1055	431.0976	99.51	C_21_H_20_O_10_	Isovitexin
27	8.226	461.109	462.1161	461.1083	97.70	C_22_H_22_O_11_	Rhamnetin 3‐rhamnoside
28	8.275	527.0497	528.0575	527.0485	89.52	C_21_H_20_O_14_S	Quercetin 3‐rhamnoside−3'‐sulfate
29	8.527	445.1139	446.1211	445.1135	99.5	C_22_H_22_O_10_	Apigenin 7‐methyl ether 4'‐glucoside
30	8.577	639.1204	640.1277	147.0114	47.59	C_27_H_28_O_18_	Nelumboside
31	8.677	511.0545	512.0619	511.0545	97.26	C_21_H_20_O_13_S	3,5,7‐Trihydroxyflavone 3‐glucoside−8‐sulfate
32	8.728	199.0249	200.0321	127.0397	99.30	C_8_H_8_O_6_	Phthalate 3,4‐cis‐dihydrodiol
33	8.878	393.0825	394.0897	199.0246	98.36	C_18_H_18_O_10_	9‐Hydroxy−4‐methoxypsoralen 9‐glucoside
34	9.029	175.0248	176.0321	115.0033	99.63	C_6_H_8_O_6_	Ascorbic acid
35	9.079	557.1292	558.1365	557.1295	98.11	C_27_H_26_O_13_	Piceatannol 4'‐galloylglucoside
36	9.129	279.1236	280.1308	139.0762	99.68	C_15_H_20_O_5_	8‐Deoxy−11,13‐dihydroxygrosheimin
37	9.681	352.176	353.1829	352.1757	73.82	C_18_H_27_NO_6_	Rosmarinine
38	10.485	285.0402	286.0475	285.0401	98.77	C_15_H_10_O_6_	Luteolin
39	10.661	350.1607	351.1679	350.1597	98.88	C_18_H_25_NO_6_	Usaramine
40	11.589	249.1105	250.1178	249.1102		C_14_H_18_O_4_	Trolox
41	11.928	385.2231	386.2303	385.2227	97.46	C_20_H_34_O_7_	Oleandolide
42	12.544	307.1908	308.1981	307.1899	83.87	C_18_H_28_O_4_	Dihydrocapsiate
43	13.196	223.077	224.0839	178.9321	83.95	C_15_H_12_O_2_	1‐Methoxy−2‐hydroxyanthracene
44	15.054	269.0452	270.0524	269.045	98.91	C_15_H_10_O_5_	Baicalein

### 3.2 Effect of APE on blood glucose concentration in DM rats

**FIGURE 2:**
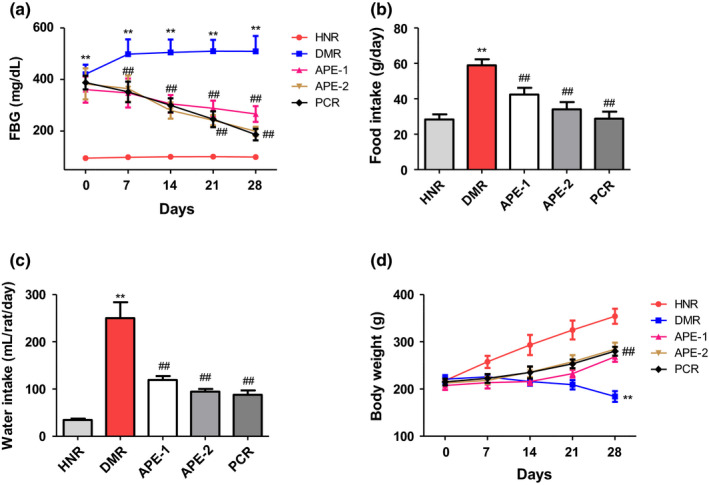
Effect of the *Acacia pennata* extract (APE) on fasting blood glucose, food intake, water intake, and body weight gain in nicotinamide/streptozotocin (NICO/STZ)‐induced diabetic rats. Values were expressed as mean ± SD (*n* = 6). Data were analyzed by analysis of variance (ANOVA) followed by Dunnett's test. **p < .001 compared with the healthy nondiabetic rats (HNR) group. ##*p* < .001, &&*p* < .01, $$*p* < .05 compared with the diabetic control rats (DMR) group

The average FBG concentration in the HNR (95.0 mg/dl) was significantly lower than the DMR rats (420.3 mg/dl) at day 0 of the treatment. At the end of the treatment period (day 28), the FBG level of the HNR was 99.3 mg/dl, which was significantly lower than the corresponding FBG level of the DMR at the same period (509.5 mg/dl). During the course of the treatment, the blood glucose level of the DMR group was consistently and significantly higher than the normal rats (Figure 1a). Whereas in diabetic rats treated with APE (100 and 400 mg/kg), there was a dose‐dependent and significant reduction of the FBG concentration to 266.1 and 198.5 mg/dl, respectively, compared to the DMR group at the end of the treatment (Figure 2a).

### 3.3 Effect of APE on body weight loss, food and water intake in DM rats

Diabetes mellitus significantly increased the food and water consumption of the DMR group when compared to the HNR group (Figure 2b‐c). Conversely, treatment of diabetic rats with APE (100 and 400 mg/kg) resulted in significant reduction in the food and water intake of the treated groups compared to the DMR (Figure 1b). Despite the significant increase in the food intake of the DMR group, there was a 17% reduction in the body weight at the end of the study, which was markedly lower than that of the HNR group. The HNR has a 62.6% increase in their body weight (Figure 1c). However, treatment with APE (100 and 400 mg/kg) significantly increased the bodyweight gain of the treated rats by 29.3 and 33.7%, respectively (Figure 2d).

### 3.4 Effect on glucose and insulin sensitivity in DM rats

**FIGURE 3:**
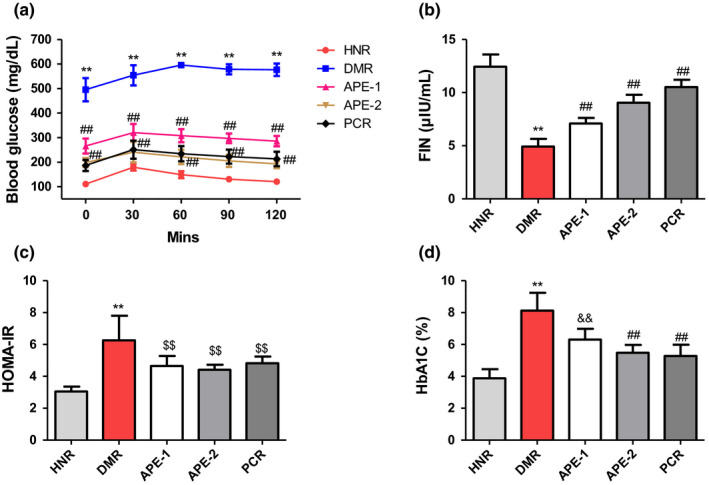
Effect of the *Acacia pennata* extract (APE) on intraperitoneal glucose tolerance test (IPGTT), fasting plasma insulin (FINS), homeostatic model assessment for insulin resistance (HOMA‐IR), and hemoglobin A1c (HbA1c) levels in nicotinamide/streptozotocin (NICO/STZ)‐induced diabetic rats. Values were expressed as mean ± SD (*n* = 6). **p < .001 compared with the healthy nondiabetic rats (HNR) group. ##*p* < .001, &&*p* < .01, $$*p* < .05 compared with the diabetic control rats (DMR) group

In the IPGTT, the blood glucose concentration of the DMR group was consistently and markedly higher at all the measured times (0, 30, 60, 90, and 120 min),compared to the corresponding values of the rats in the HNR group. Whereas, the glucose sensitivity was significantly reduced in the diabetic rats treated with APE from 60 to 120‐min postglucose administration compared to the DMR group (Figure 3a). Additionally, the DMR rats showed significantly reduced FINS levels compared to the HNR group, but significantly improved in APE‐treated groups (Figure 3b). Furthermore, HOMA‐IR and HbA1c were observed to be significantly increased in the diabetic control group, while treatment of diabetic rats with APE markedly decreased HOMA‐IR and HbA1c compared to the DMR group (Figure 3c‐d).

### 3.5 Effect on the serum biochemical parameters in DM rats

**FIGURE 4:**
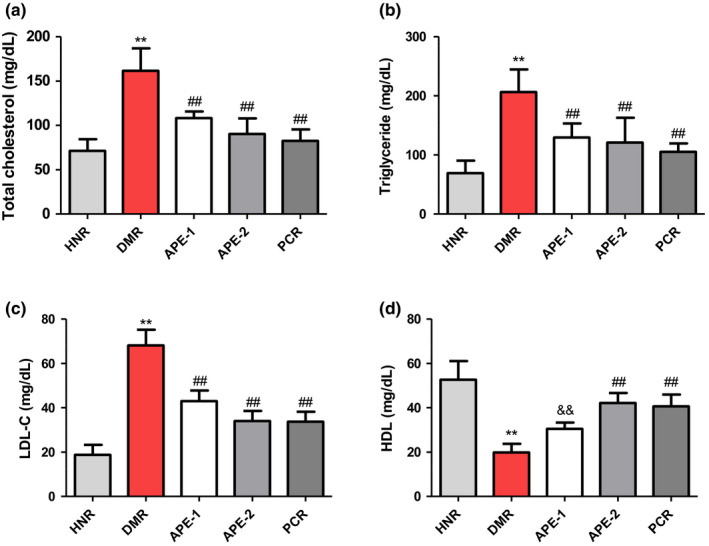
Effect of the *Acacia pennata* extract (APE) on serum total cholesterol (TC), triglycerides (TG), low‐density lipoprotein cholesterol (LDL‐C), and high‐density lipoprotein (HDL) test in nicotinamide/streptozotocin (NICO/STZ)‐induced diabetic rats. Values were expressed as mean ± SD (*n* = 6). **p < .001 compared with the healthy nondiabetic rats (HNR) group. ##*p* < .001, &&*p* < .01, $$*p* <.05 compared with the diabetic control rats (DMR) group

**FIGURE 5:**
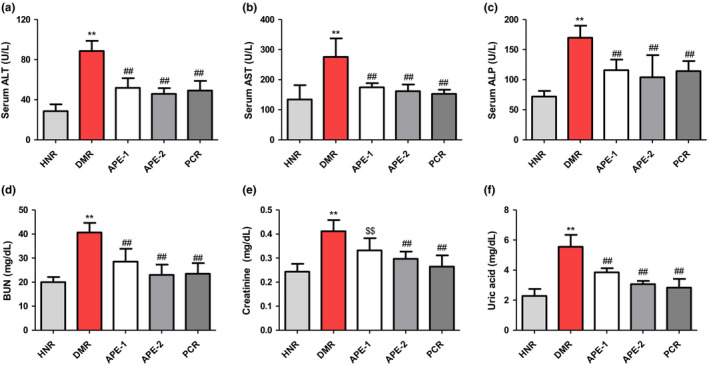
Effect of the *Acacia pennata* extract (APE) on serum alanine transaminase (ALT), aspartate transaminase (AST), alanine phosphatase (ALP), blood urea nitrogen (BUN), creatinine, and uric acid in nicotinamide/streptozotocin (NICO/STZ)‐induced diabetic rats. Values were expressed as mean ± *SD* (*n* = 6). **p < .001 compared with the healthy nondiabetic rats (HNR) group. ##*p* <.001, &&*p* <.01, $$*p* <.05 compared with the diabetic control rats (DMR) group

The results presented in Figure 4 indicate that the serum lipids including total cholesterol, triglycerides. and low‐density lipoprotein cholesterol (LDL‐C) levels in the DMR group were drastically increased (161, 206, and 68.1 mg/dl, respectively), while HDL was reduced compared to the corresponding values in the HNR group (71.3, 69, and 18.8 mg/dl, respectively). Additionally, the serum hepatorenal biomarkers, including ALT, AST, ALP, BUN, creatinine, and uric acid, were significantly increased relative to the HNR group (Figure 5a‐f). Conversely, treatment of diabetic rats with APE significantly ameliorated these serum biochemical alterations when compared to the DMR group (Figures 4 and 5).

### 3.6 Effect on pancreas histology in DM rats

**FIGURE 6:**
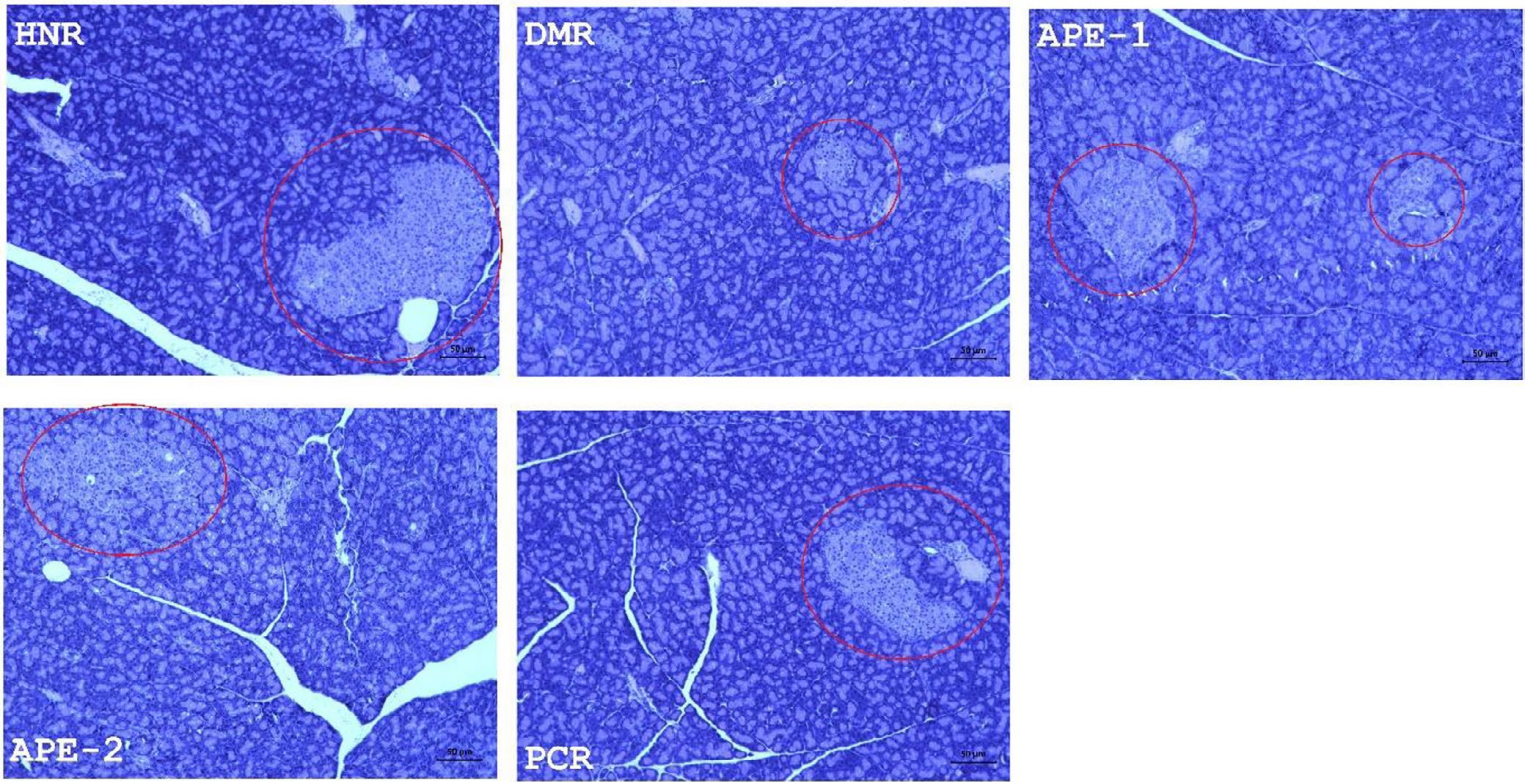
Representative pancreatic photomicrographs of hematoxylin and eosin (H&E)‐stained sections (magnification 40 × 400). Red circle indicates the islets of Langerhans. APE, *Acacia pennata* extract; DMR, diabetic control rats; HNR, healthy nondiabetic rats

The representative H&E‐stained pancreas of the HNR group indicated significant number and size of the islet of Langerhans, compared to the DMR group with a reduced islet size as well as necrotic acinar cells (Figure 6a,b). The representative images from the pancreas of APE (100 and 400 mg/kg) and metformin‐treated groups showed restored islet of Langerhans cellularity, as indicated by increased size of the islets as well as improved pancreas acinar cells (Figure 6).

### 3.7 Effect on oxidative stress biomarkers in DM rats

As shown in Table 2, DM markedly increased pancreatic MDA level in the DMR group compared with the HNR. However, the administration of APE and metformin significantly lowered MDA levels in the pancreas compared with the DMR group. The modulatory effects of APE treatment on antioxidant capacity in diabetic rats are also portrayed in Table 2. In line with the MDA results, the activities of antioxidant enzyme biomarkers including SOD, GPx, and CAT were significantly declined in the pancreatic tissues of the DMR group. In contrast, the rats treated with APE and metformin displayed significant increases in GPx, SOD, and CAT activities in the pancreatic tissues of the treated rats compared to the DMR group (Table 2).

**TABLE 2:** Effect of the *Acacia pennata* extract (APE) on pancreatic oxidative stress and antioxidant parameters nicotinamide/streptozotocin (NICO/STZ)‐induced diabetic rats

Groups	MDA (nmol/mg prot)	GPx (U/mg prot)	SOD (U/mg prot)	CAT (U/mg prot)
HNR	10.47 ± 1.45	250.91 ± 21.28	129.91 ± 10.34	78.61 ± 9.77
DMR	35.01 ± 5.10*	119.75 ± 17.66*	61.61 ± 8.74*	31.14 ± 6.50*
APE−1	22.86 ± 3.48**	170.51 ± 17.33**	88.8 ± 8.12**	55.61 ± 5.02**
APE−2	16.13 ± 3.48**	192.83 ± 9.77**	106.86 ± 6.31**	65.38 ± 5.05**
PCR	17.61 ± 3.03**	184.4 ± 14.54**	107.78 ± 8.60**	68.3 ± 6.24**

Abbreviations: APE‐1, diabetic rats administered 100 mg/kg of APE; APE‐2, diabetic rats administered 400 mg/kg of APE; CAT, Catalase; DMR, diabetic control rats; GPx, Glutathione peroxidase; HNR, healthy nondiabetic rats; MDA, Malonaldehyde; PCR, positive control rats; SOD, Superoxide dismutase.

Values were expressed as mean ± SD (*n* = 6). Data were analyzed by the analysis of variance (ANOVA) followed by Dunnett's test.

*p < .001 compared with the HNR group.

**p < .001 compared with the DMR group.

## 4 DISCUSSION

Impaired insulin resistance/secretion is the major cause of type 2 diabetes mellitus and it is often characterized by impairment in β‐cell function, protein, lipids, and carbohydrate metabolism (DeFronzo et al., 2015; Olatunji et al., 2018). The increase in the incidence and prevalence of DM on the global scale as well as the morbidity/mortality rate associated with comorbidities in diabetes coupled with the unpleasant side effects of contemporary synthetic antidiabetic drugs has fueled the drive for the discovery of alternative sources for preventing or treating diabetes mellitus (Artasensi et al., 2020; Babar et al., 2019; Williams et al., 2020). As such exploring medicinal plants as therapeutics for diabetes may be a viable option for counteracting the problems relating to adverse effects, cost, and availability. The present study is the first to demonstrate the antidiabetic effects of APE. The results indicated that APE alleviated diabetes‐associated pancreato‐hepatorenal dysfunction by decreasing oxidative stress, glucose, and insulin sensitivity in NICO/STZ‐induced diabetic rats. Diabetic rats presented significantly increased FBG, serum lipids (TC, TG, and LDL‐C), liver and kidney function (ALT, ALP, AST, BUN, and creatinine), reduced serum FINS, and antioxidant enzymes (GPx, SOD, and CAT) as well as significant increase in the lipid peroxidation (MDA) in the pancreas of NICO/STZ‐induced DM rats. These results agreed with previous reports that indicated that diabetic rats presented impaired kidney, liver, and pancreatic function as well as increased oxidative stress in vital organs (Gao et al., 2021; Makinde et al., 2020; Olatunji et al., 2017). The results of the present study showed that treatment with APE significantly increased insulin secretion, reduced glucose sensitivity deficits, lipid profiles, oxidative stress, ameliorated β‐cell function, and improved islet structure in NICO/STZ‐induced DM rats.

Under normal physiological state, the body converts excessive glucose in the blood to glycogen through the stimulation of insulin. Whereas, in type 2 diabetes, insulin secretion/sensitivity is impaired resulting in reduced production of insulin which invariably leads to decrease in hepatic glucose conversion to glycogen, cumulating in a high level of glucose in the blood. Additionally, insulin is also responsible for the activation of lipoprotein lipase, a vital enzyme responsible for removing circulating triglycerides in the blood. As such, the insufficiency in insulin concentration experienced in DM results in low circulating lipoprotein lipase enzymes and high lipid accumulation in the blood. These conditions have been linked to a high incidence of cardiovascular diseases in diabetic patients (Mollica et al., 2017; Tan et al., 2006). Thus, regulating insulin secretion can improve glucose conversion, synthesis of lipids, and blood glucose level. The result obtained from this study indicated that APE markedly increased insulin secretion, islets cellularity, and ameliorated pancreatic architecture in diabetic rats, which in turn led to decrease in the blood glucose and lipid profile concentrations. Treatment with APE resulted in significant reduction in blood glucose, TG, TC, and LDL‐C levels.

The oral administration of APE significantly altered diabetes‐induced hepatorenal dysfunction, as indicated by a reduction in functional biomarkers of the liver (ALT, AST, and ALP) and kidney (BUN, uric acid, and Scr). Diabetes has been strongly implicated in liver and kidney abnormalities. The release of markers of hepatotoxicity including ALP, AST, and ALT into the blood suggests hepatic injury (Adefegha et al., 2014). The liver is critically involved in several physiological processes including insulin clearance, glucose regulation, lipid and carbohydrate metabolism, and deficiencies in insulin function can cause liver abnormalities due to gluconeogenesis, resulting in increased serum liver enzymes (Ghosh & Suryawanshi, 2001). Furthermore, diabetic rats showed increased levels of typical biomarkers of renal injury including high levels of serum BUN, urea, and creatinine which are consistent with previous studies (Kishore et al., 2017; Kpemissi et al., 2020; Zhang et al., 2020). Treatment of diabetic rats with APE resulted in a dose‐dependent amelioration of serum hepatorenal dysregulated biomarkers. APE significantly decreased BUN, creatinine, uric acid, AST, ALT, and ALP levels.

Hyperglycemia plays a vital role in the generation of reactive oxygen and oxidative stress. Indeed, accumulating lines of evidence have showcased the prominent role of oxidative stress in the development and progression of comorbidities in diabetes (Marvibaigi et al., 2021). Increased oxidative stress has also been implicated in insulin resistance and β‐cell injury resulting in a decline in insulin secretion (Gao et al., 2021). In agreement with previous reports, this study demonstrated oxidative imbalance in the redox status of the pancreas of DM rats, as observed by decreased pancreatic antioxidant activities including GPx, SOD, and CAT, as well as increased levels of MDA. These antioxidants are present in cells, organs, and tissues and they assist in preventing oxidative damage caused by free radicals (An et al., 2020; Samadi‐Noshahr et al., 2020; Song et al., 2021). The oral administration of APE significantly improved the activities of GPx, CAT, and SOD in the pancreas of diabetic‐treated rats. In addition, the elevated level of MDA was markedly improved upon APE treatment, suggesting the antioxidant effect of APE in diabetic tissue injury.

According to previous studies, the extracts obtained from *A. pennata* showed robust antioxidant activity and markedly protected against acetaminophen‐induced reactive oxygen species (ROS) generation in liver tissues of treated rats (EL‐Taher et al., 2021). Consistently, the results from our experiments showed that APE demonstrated antidiabetic and antioxidant properties, suggesting its favorable alleviative effects on ROS and oxidative damage in DM. The result on the tentative identification of bioactive compounds using UPLC‐ESI‐MS/MS analysis showed the presence of different polyphenolic compounds in APE, majorly flavonoid glycosides including cynaroside A, rutin, glucocaffeic acid, patuletin 3‐rhamnoside‐7‐(3''',4'''‐diacetylrhamnoside), Saponarin, luteolin 3'‐methyl ether 7,4'‐dixyloside, isovitexin, rhamnetin 3‐rhamnoside, nelumboside, quercetin 3‐rhamnoside‐3'‐sulfate, and apigenin 7‐methyl ether 4'‐glucoside. Several nonglycosylated phenolic derivatives, such as chlorogenic acid, 3‐O‐caffeoyl‐4‐O‐methylquinic acid, trolox, luteolin, and baicalein, were also identified. The identification of these classes of compounds in APE is consistent with those of previous studies. For instance, EL‑Taher et al. identified the presence of nine flavonoid glycosides (including kaempferol 3,7‐di‐O‐hexoside, apigenin 6,8‐di‐*C*‐hexoside, luteolin‐6‐*C*‐pentoside‐8‐*C*‐hexoside, and apigenin‐6‐*C*‐hexoside‐8‐*C*‐pentoside) from the leaves of *A. pennata* using LC–ESI–MS analysis (EL‐Taher et al., 2021). In another study, 11 flavonoid glycosides were reported to have been isolated from the aerial parts of *A. pennata* (Kim et al., 2015). Among the compounds tentatively identified in our study, cynaroside A, rutin, isovitexin, luteolin, and baicalein have been reported as good antidiabetic and antioxidant agents (Fang et al., 2020; Ghorbani, 2017; Wang et al., 2021; Wei et al., 2017). Numerous studies have indicated that diabetes‐induced oxidative damages can effectively be mitigated by polyphenolic constituents due to their ability to interfere in the pathophysiology of the disease (Cao et al., 2019; Domínguez Avila et al., 2017; Solayman et al., 2016; Sun et al., 2020). As such, the synergistic hypoglycemic and antioxidant properties of these metabolites may possibility account for the ameliorative effects of APE on the diabetic rats.

## 5 CONCLUSION

Taken together, the results presented in this study portrayed the therapeutic effect of APE against NICO/STZ‐induced DM. This is evidenced by the ability of APE to alleviate glucose and insulin homeostasis, ameliorate metabolic derangement including dyslipidemia, hepatorenal dysfunction, and restore pancreatic oxidative stress and β‐cell function in the treated diabetic animals. These findings may enhance a better understanding of the therapeutic effect of *A. pennata* on diabetes and the possible development of the plant as for future antidiabetic application. However, further molecular studies are needed to further shed deeper insight into the antidiabetic mechanism of APE.

## CONFLICT OF INTEREST

None declared by the authors.

## Data Availability

The datasets generated during and/or analyzed during the current study are available from the corresponding author upon reasonable request.
